# Apatinib as non-first-line treatment in patients with Intrahepatic Cholangiocarcinoma

**DOI:** 10.7150/jca.53482

**Published:** 2021-01-10

**Authors:** Jinzhu Mao, Xu Yang, Jianzhen Lin, Xiaobo Yang, Dongxu Wang, Lei Zhang, Yi Bai, Jin Bian, Junyu Long, Fucun Xie, Hanchun Huang, Xinting Sang, Shuguang Chen, Haitao Zhao

**Affiliations:** 1 Department of Liver Surgery, Chinese Academy of Medical Sciences and Peking Union Medical College (CAMS & PUMC), Peking Union Medical College Hospital, Beijing, China.; 2 Pancreas Center, The First Affiliated Hospital of Nanjing Medical University; Pancreas Institute, Nanjing Medical University, Nanjing 210000, China.; 3 Department of General Surgery, Chinese Academy of Medical Sciences and Peking Union Medical College (CAMS & PUMC), Peking Union Medical College Hospital, Beijing, China.

**Keywords:** cholangiocarcinoma, anti-angiogenesis therapy, Apatinib, efficacy, safety

## Abstract

**Purpose:** There is limited standard treatment for patients with advanced cholangiocarcinoma after refractory of chemotherapy. Apatinib is a tyrosine kinase inhibitor targeting VEGFR-2, which exhibited broad-spectrum antitumor activities in previous studies. We aim to evaluate the efficacy and safety of apatinib as non-first-line treatment in patients with advanced cholangiocarcinoma.

**Methods:** This was a prospective open-label phase II trial (NCT03251443). Patients with pathology-confirmed cholangiocarcinoma after prior systemic therapy were enrolled. Participants were treated with apatinib 500 mg orally once daily. The primary end point was overall response rate (ORR).

**Results:** Between August 8, 2017 and November 13, 2018, 30 patients participated in this study, and 26 patients received apatinib treatment except 4 patients withdrew consent before the first dosage. For full analysis set, the ORR was 11.5% and the disease control rate was 50.0%. 3 patients (11.5%) achieved partial response and no patients achieved complete response. The median progression free time was 2.0 (95% CI: 0.7-3.3) months and median overall survival was 9. 0 (95% CI: 4.6-13.4) months. The most common adverse events of any grade were fatigue (80.8%), hypertension (73.1%) and decreased appetite (38.5%). Grade 3 adverse events occurred in 23.1% patients and no grade 4 adverse events occurred. The most common grade 3 adverse events were hypertension (23.1%) and elevated transaminase (11.5%).

**Conclusion:** Apatinib as non-first-line monotherapy has potential therapeutic efficacy in patients with advanced cholangiocarcinoma.

## Introduction

Cholangiocarcinoma (CCA) is a malignant tumor arising from the epithelial cell in the second and upper degree bile ducts, including intrahepatic cholangiocarcinoma (iCCA) and extrahepatic cholangiocarcinoma (eCCA) 1. iCCA account for approximately 10-20% of all CCAs while eCCA account for approximately 80-90% 2. Mortality rates were around 1-2/100,000 for iCCA and below 1/100,000 for eCCA in most countries 3. iCCA accounts for 10-15% primary liver cancer as the second most common primary liver cancer 4 and it has become the most common cause of all the primary liver tumor death 5. Patients with CCA usually present with advanced disease at diagnosis and approximately 20% cases are eligible to receive surgery treatment 6. For the patients with advanced CCA, combination of gemcitabine and cisplatin is recommended as the first line treatment regimen, which achieved a median progressive free survival (PFS) of 8 months and median overall survival (OS) of 11.7 months 7. After failure with first line chemotherapy, fluoropyrimidine-based chemotherapy is considered as the second line treatment, such as 5-fluorouracil (5-FU) and capecitabine, which combined with gemcitabine or cisplatin had been assessed in multiple phase II trials 8. Moreover, emerging outcomes from the ABC-06 trial demonstrated that patients could achieve survival benefits from second-line mFOLFOX (5-FU plus oxaliplatin) with active symptom control (ASC) 9. Other options include molecular targeted therapy, immune checkpoint blockade therapy and locoregional therapy, such as radiation and transarterial chemoembolization (TACE), however, there are limited evidences from clinical trials currently 5, 8. Due to low response rate, treatment toxicities intolerance and high recurrence rates, five-year relative survival rates for CCA is approximately 5% 2, ranging from 2% to 15% for iCCA and from 2% to 30% for eCAA, respectively 6. Therefore, therapeutic regimens are urgently required to achieve better efficacy to improve the survival for patients with CCA.

Angiogenesis is a hallmark process in tumor growth and metastasis, and anti-angiogenesis has become a promising strategy in cancer treatment 10. Vascular endothelial growth factor (VEGF) and its receptors (VEGFRs) including VEGFR-1, VEGFR-2 and VEGFR-3 are responsible in tumor angiogenesis 11. In CCA, overexpression of VEGF was observed in 53.8% cases and involved in intrahepatic and haematogenic metastasis 12, while the expression of VEGFR was related with the proliferation and differentiation of CCA 13. Thus, inhibiting of VEGF/VEGFR signaling may be a potential strategy for advanced CCA treatment. However, whether anti-angiogenesis therapy can improve the efficacy in patients with advanced CCA and what extent the improvement can achieve remains unclear.

Apatinib is an oral small-molecule tyrosine kinase inhibitor (TKI) which plays function in anti-angiogenesis by selectively inhibiting VEGFR-2. It has been reported in a series of clinical trials that apatinb demonstrated encouraging anti-tumor activities with tolerable toxicities in multiple advanced cancers, such as gastric cancer 14, colorectal cancer 15, ovarian cancer 10, 16 and breast cancer 17, 18. In a phase III study, apatinib monotherapy significantly improved PFS and OS compared with the placebo group (PFS 2.6 months versus1.8 months, OS 6.5 months versus 4.8 months) in patients with advanced gastric cancer refractory to chemotherapy 14. A retrospective cohort study including 32 patients with unresectable or relapsed liver cancer showed that apatinib monotherapy as second line treatment achieved an ORR of 16%, a median PFS of 5.0 months for HCC and a median PFS of 3.0 months for iCCA 19. However, whether apatinib can inhibit tumor growth by inhibiting VEGF/VEGFR signaling pathway and improve the outcomes in patients with iCCA needs prospective interventional clinical investigation.

In this phase II trial, we aimed to prospectively and preliminarily investigate the therapeutic efficacy and safety of apatinib as an alternative second-line treatment option in patients with refractory or metastatic CCA.

## Methods

### Study population

Patients with histologically proven and measurable unresectable or metastatic primary iCCA were eligible. Other inclusion criteria included age ≥18 years; progressive disease after at least one lines of systemic therapy for advanced cholangiocarcinoma; Eastern Cooperative Oncology Group (ECOG) performance status 0-2; life expectancy of at least 12 weeks; with enough function in bone marrow hematopoiesis and adequately controlled liver and kidney function.

Key exclusion criteria included previous locoregional therapy within 4 weeks prior to enrollment; prior other VEGFR-targeted therapies; with central nervous system metastases; active bleeding in gastroenterological tract; uncontrolled blood pressure >140mmHg (systolic) or >90mmHg (diastolic); clinically significant cardiac arrhythmia, stroke, myocardial infarction; and pregnancy or lactation. All patients provided written informed consent before study participation. The protocol was approved by the institutional review board (IRB) of Peking Union Medical College Hospital (PUMCH).

### Study design

This is a single-arm, phase 2 study to preliminarily evaluate objective response rate (ORR) and tolerability of apatinib in patients with advanced intrahepatic cholangiocarcinoma. Participants were treated by apatinib (500mg orally daily on a continuous schedule) for 28-days per cycle. Dose reductions were permitted down to 250mg daily. The endpoints included the ORR, the disease control rate (DCR), progression-free survival (PFS), overall survival (OS) and safety. The study was initiated at August 8, 2017 and was completed at May 8, 2019. This clinical trial was registered in ClinicalTrials.gov (identifier: NCT03251443).

### Efficacy and safety assessments

Patients were evaluated for a response radiographically with enhanced computed tomography (CT) or magnetic resonance imaging (MRI) and serologically with carbohydrate antigen (CA199) levels every 6 weeks. The criteria of therapeutic response referred to the Response Evaluation Criteria in Solid Tumors (RECIST, version 1.1) by an independent radiological review. Patients continued treatment until confirmed progressive disease (PD), unacceptable toxicity, or the withdrawal of consent.

Safety and tolerability were assessed all through the treatment cycles according to Common Terminology Criteria for Adverse Events (CTCAE) version 4.03. Patients were routinely monitored for safety every 2 weeks in each cycle. Safety evaluations majorly included ECOG evaluation, blood pressure measurements, completed blood counts, blood chemistries, coagulation studies, urinalyses and a physical examination.

### Statistical analysis

In the setting of the initial sample size, with reference to the sample size of majority of phase II trials in biliary tract cancer, under an expected ORR of 20%, a certainty of 80%, a single type I error of 0.05 and a placebo-controlled ORR of 5%, we estimated sample size as 28 patients by using an online web-tool (https://stattools.crab.org/Calculators/oneArmBinomial.html). Considering the leakage rate of 10%, we decided to enroll a total of 30 patients. All participants who had received at least one dose of apatinib were included for the analysis. 30 patients with advanced cholangiocarcinoma signed the informed consent while 4 patients of them were excluded because of withdrawing the consents before the first dose of apatinib. Therefore, 26 patients were enrolled in full analysis set (FAS). ORR and DCR were calculated for the proportion of patients with objective responses and controlled disease. Cases with incomplete follow-up or without adequate radiological evaluations were censored at the date on which they were last documented to be progression-free. PFS and OS were estimated with the Kaplan-Meier method. To compare the individual variables, the χ2 test, Fisher's exact test, and Spearman's ρ coefficient test were performed as appropriate. A two-tailed p-value <0.05 was considered significant. All statistical analyses were performed by SPSS version 24.0 (IBM, Chicago, IL).

## Results

### Patient Demographics

From August 8, 2017 to November 13, 2018, a total of 30 CCA patients participated in this trial and 26 patients with refractory iCCA were enrolled in full analysis set (FAS). 4 patients were excluded for withdrawing the consents before the first dose of apatinib. In the FAS, 26 patients were available for assessment of efficacy and safety. The detail information of baseline characteristics is listed in Table 1. Of all the 26 patients, the median age was 56 years old, ranging from 35 to 77 years old. Men were more common than women (male: female=1.9:1). The ECOG performance status score was 0-1 and the Child-Pugh score for liver function was grade A for all patients. According to the 7th AJCC cancer staging, 5 patients (19.2%) were clinically stage III and 21 patients (80.8%) were clinically stage IV. In total, 18 patients (69.2%) had received surgical treatment for primary tumor control and 16 patients (61.5%) patients had received locoregional treatment, including TACE, radiofrequency ablation (RFA) and radiotherapy. 10 patients (26.7%) progressed from gemcitabine-based or platinum-based chemotherapy. 2 patients (7.7%) received tyrosine kinase inhibitor treatment, including sorafenib (1 case) and afatinib (1 case). 2 patients (7.7%) received immune checkpoint inhibitor treatment (anti-PD1 antibody). 7 patients (26.9%) had a history of HBV infection and 1 patient (3.8%) had a history of HCV infection.

### Efficacy

Overall, all the 26 patients in FAS received at least one dose of apatinib with initial dose of 500 mg daily. Due to drug-related AEs intolerance, dose reduction from 500 mg daily to 250 mg daily occurred in 13 patients. Among these 13 patients, 8 patients were well tolerant and continued treatment, while the other 5 patients quit medication for toxicity intolerance. Totally, 18 patients received medication for more than 1 months. The follow-up continued until all the patients met OS, and the median duration of follow-up was 10 months.

**Figure 1:**
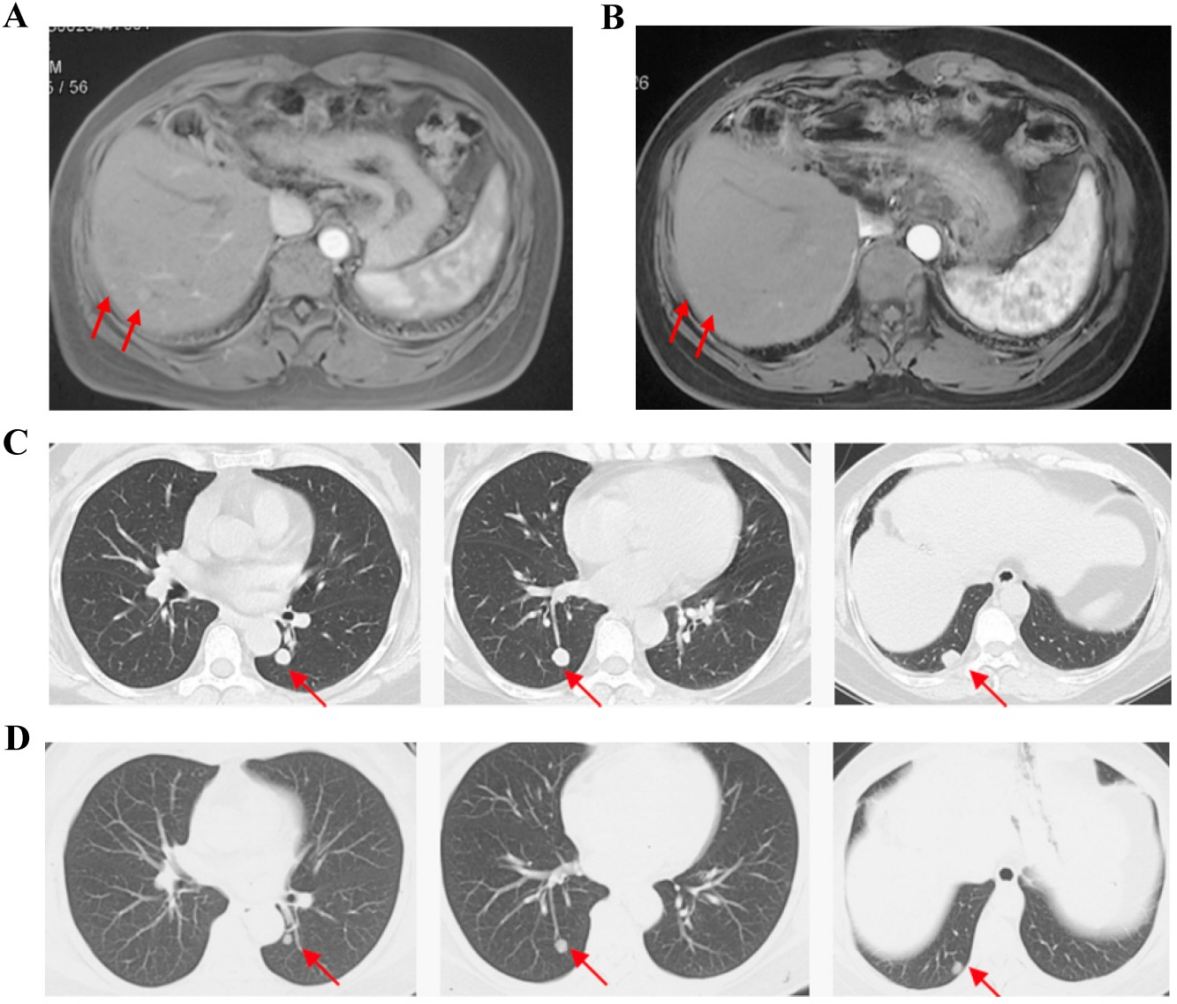
Cases Show partial response with apatinib monotherapy. Case 1, F/35y, relapsed with 2 foci in liver after surgery and TACE (a), achieved partial response after receiving apatinib 500 mg/d for 2 months and the response duration was 2 months (b). Case 2, F/54y, relapsed with 3 metastatic foci in lung after surgery, chemotherapy and TACE (c), achieved partial response after apatinib 500 mg/d for 3.5 months and the response duration was 2 months (d).

**Figure 2:**
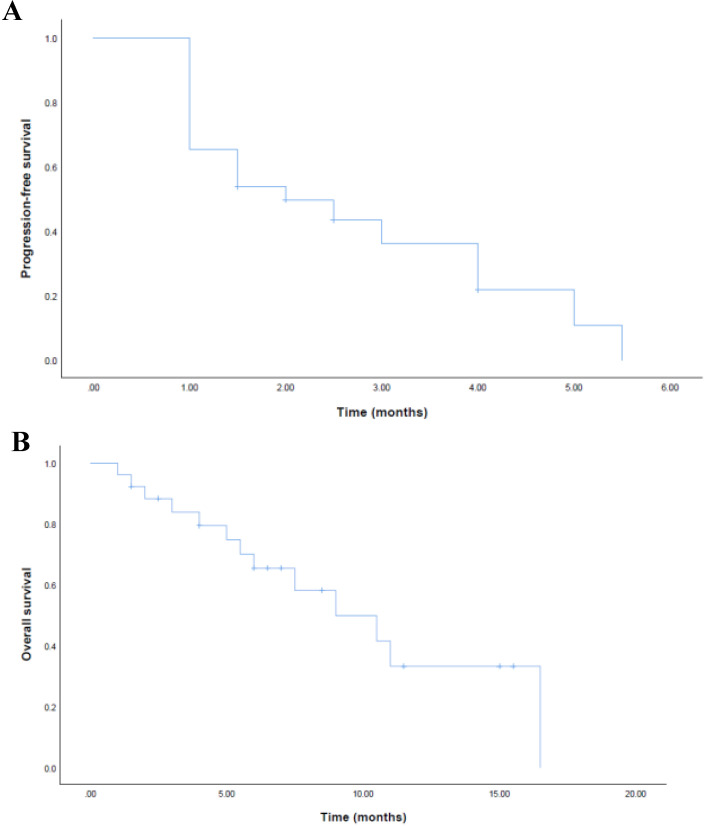
Kaplan-Meier estimates of progression-free survival (a) and overall survival (b) for FAS.

For FAS, the ORR was 11.5% and the DCR was 50.0% (Table 2). No patient achieved complete response (CR). 3 patients achieved partial response (PR) and the mean time of response duration was 2 months (Figure 1). 10 patients achieved stable disease (SD) and the mean time of response duration was 2.6 months, ranging from 1.5 months to 5 months. Kaplan-Meier curves estimated that the median PFS was 2.0 months (95% CI, 0.7-3.3) and the median OS was 9.0 months (95%CI, 4.6-13.4) (Figure 2).

### Safety

All the AEs related with apatinib treatment in FAS were summarized in Table 3. Generally, apatinib treatment associated AEs appeared in all the treated participants while no treatment related death happened. Therapeutic termination due to AEs occurred in 13 patients. Grade 3 AEs occurred in 6 patients (23.1%) and no grade 4 AEs were observed. The most common grade 3 AEs were hypertension (23.1%) and elevated transaminase (11.5%), other grade 3 AEs included fatigue, vomiting and hematologic abnormality. Furthermore, 3 patients (11.5%) appeared hematologic toxicities including anemia, neutropenia, thrombocytopenia and thrombosis. Considering AEs intolerance and safety issues, therapeutic termination occurred in 5 patients (19.2%) and a dose reduction from 500mg daily to 250mg daily was subsequently started in 8 patients (30.8%). Of all the AEs, the most common AEs were fatigue (80.8%), hypertension (73.1%) and decreased appetite (38.5%), and other common AEs included elevated aminotransferase, nausea, rash and hand-foot syndrome.

## Discussion

The present study prospectively evaluated the efficacy and safety of apatinib monotherapy as second-line and above treatment in patients with refractory CCA. In this phase II trial, we observed that apatinib monotherapy showed promising anti-tumor activity with controllable toxicity profiles in the treatment-tolerable populations. Of all the 26 patients enrolled in this study, the median PFS was 2.0 (95% CI: 0.7-3.3) months and the median OS was 9.0 (95% CI: 4.6-13.4) months. And the ORR achieved 11.5% and the DCR achieved 50.0%, respectively. The obstacle of treatment duration of apatinib in CCA patients might be the drug-related AEs tolerability. Although there was no grade 4 AEs, grade 3 AEs occurred in 23.1% patients, which resulted in medication termination or dose reduction and this might affect the application of apatinib treatment in advanced CCA.

Due to the limited survival improvement of second-line chemotherapy, more and more second-line treatment strategies have been explored and assessed in a series of studies during the last decade, including combinational systemic chemotherapy, molecular targeted drugs and immune checkpoint inhibitors. However, only second-line mFOLFOX has been demonstrated to improve the survival benefits (mFOLFOX+ASC versus ASC: HR 0.69, 95% CI 0.50-0.97, p=0.031) for patients with unresectable biliary tract cancers who were previously treated with cisplatin/gemcitabine 9. Recently, a single-arm phase 2 trial enrolling 30 patients reported that fluorouracil, leucovorin, irinotecan plus oxaliplatin (FOLFIRINOX) achieved median PFS of 6.2 months and median OS of 10.7 months in patients with advanced biliary tract cancers intolerant or progressed from gemcitabine plus cisplatin 20. For molecular targeted therapy as second-line therapy, only ivosidenib (a small-molecule targeted inhibitor of IDH1) significantly improved median PFS compared with placebo (2.7 months vs 1.4 months, hazard ratio 0.37, one-sided p<0.0001) in patients with previously treated IDH1-mutant cholangiocarcinoma 21. Notably, various retrospective analyses and clinical case reports suggest apatinib has promising activity and manageable toxicity for the pretreated patients with advanced biliary tract cancers, with ORR around 15% and median PFS around 3.0 months 19, 22-24. In this study, apatinib monotherapy preliminarily showed an underlying anti-tumor activity in patients with advanced CCA, which confirmed the potential therapeutic value of anti-angiogenesis strategy and encouraged further exploration of anti-angiogenesis drugs in chemotherapy refractory CCA. Actually, other anti-angiogenesis molecular targeted drugs, such as regorafenib 25 and lenvatinib 26, had been explored and demonstrated similar anti-angiogenesis efficacy as apatinib in patients with advanced CCA, especially for the patients failed with gemcitabine-based chemotherapy. Therefore, our trial has provided valuable experience about apatinib application for clinical trials in the future.

Fatigue, hypertension and decreased appetite were the most common AEs related with apatinib treatment, consistent with the results reported in previous studies in HCC 27, ovarian cancer 16, gastric cancer 14 and breast cancer 17, 18. Although there were no grade 4 AEs, grade 3 AEs occurred in 23.1% patients in FAS and the most frequently reported grade 3 AEs were hypertension (23.1%) and elevated transaminase (11.5%). Of the 2 patients developed hematologic toxicities, 1 patient appeared grade 3 anemia and thrombocytopenia, leading to medication discontinuation. All the AEs caused by apatinib treatment were manageable and no treatment-related death happened in this study. However, the incidence of grade 3 or higher AEs was unexpectedly higher compared with previous studies and resulted in high rate of therapeutic termination of 13 (50.0%) cases in our study. Although the anti-tumor activity of apatinib was promising in this trial, high incidence of AEs may hinder the clinical application of apatinib in advanced CCA. We proposed that high incidence of severe AEs from apatinib treatment might be associated with enrollment of a large portion of more advanced patients. One the one hand, the liver function of these advanced patients tended to decline after disease progressed from previous chemotherapy or locoregional treatment, which induced the intolerance of sequential apatinib treatment. On the other hand, most patients with CCA had metabolic disorders, such as cholestatic jaundice, inflammation of biliary tracts and gastrointestinal dysfunction, which affected the pharmacokinetics of apatinib and resulted in dose intolerance. Moreover, the initial dose and medication cycle of apatinib require further exploration in clinical practice. In another dose escalation and expansion study assessing SHR-1210 (anti-PD-1 antibody) plus apatinib for advanced HCC, gastric or esophagogastric junction cancer 28, fewer AEs occurred in the group received apatinib 250 mg daily than that received 125 mg or 500 mg daily, which recommended that the maximum tolerated dose for apatinib was 250 mg daily. However, response and survival improvement were observed after escalating from 125 mg to 250 mg and this result showed that clinical efficacy might positive related with dose escalation in a safe range. Whether initiation dose with 250 mg daily instead of 500 mg daily is more rational in advanced cholangiocarcinoma remains further investigation. Therefore, balancing dose and AEs is important and it is essential to combine best supportive care or ASC when adopting apatinib as second-line treatment. Timely surveillance and management of AEs should be encouraged and symptomatic treatment or dose adjustment is necessary to control the AEs during apatinib administration.

There were some limitations in our study. First, this study was a single arm, single-center trial and no control arm was administrated. And we could not rule out that patients with different treatment background were enrolled, especially a large portion of more advanced patients. Second, the sample size was small in the present study. Third, a portion of patients discontinued medication and quit the trial before efficacy assessment because of high incidence and intolerance of AEs, which might affect the real efficacy. Last, heterogeneous responses were presented in this trial and this was limited by lack of suitable biomarkers of anti-angiogenic agents. Thus, further study with large sample sizes should include biomarkers investigation to predict whether patients will benefit from apatinib treatment or identify the patients most likely to be benefited from apatinib treatment.

In conclusion, this study demonstrated the apatinib potential anti-tumor activity of as anti-angiogenesis agent in patients with advanced CCA who were tolerable for the treatment.

## Figures and Tables

**Table 1:** Patient demographics and clinical characteristics in full analysis set (n=26)

Characteristics	No. (%)
Median age (range)	56 (35-77)
**Sex**	
Male	17 (65.4)
Female	9 (34.6)
**ECOG performance status**	
0	17 (65.4)
1	9 (34.6)
**Child-Pugh score**	
A	26 (100)
**Clinically stage**	
III	5 (19.2)
IV	21 (80.8)
Primary tumor site (intrahepatic)	26 (100)
**Previous therapy**	
Surgery	18 (69.2)
TACE	12 (46.2)
RFA	8 (30.8)
Radiotherapy	3 (11.5)
Chemotherapy	10 (38.5)
Targeted therapy	2 (7.7)
Immunotherapy	2 (7.7)
HBV infection	7 (26.9)
HCV infection	1 (3.8)
Hypertension	6 (23.1)

**Table 2:** Efficacy analysis for full analysis set (n=26)

Survival	Months (95% CI)
mPFS	2.0 (0.7-3.3)
mOS	9.0 (4.6-13.4)
**Response**	**No. (%)**
CR	0 (0)
PR	3 (11.5)
SD	10 (38.5)
PD	13 (50.0)
ORR	3 (11.5)
DCR	13 (50.0)

mPFS, median progression-free survival; mOS, median overall survival; CR, complete response; PR, partial response; SD, stable disease; PD, progression disease; ORR, overall response rate; DCR, disease control rate.

**Table 3:** Possible apatinib treatment-related adverse events

Adverse Events	No. (%)			
	Any Grade	1	2	3
Hypertension	19 (73.1)	5 (19.2)	8 (30.8)	6 (23.1)
Elevated aminotransferase	7 (26.9)	4 (15.4)	0 (0)	3 (11.5)
Fatigue	21 (80.8)	10 (38.5)	9 (34.6)	2 (7.7)
Nausea	10 (38.5)	6 (23.1)	4 (15.4)	0 (0)
Vomiting	3 (11.5)	2 (7.7)	0 (0)	1 (3.8)
Diarrhea	2 (7.7)	0 (0)	2 (7.7)	0 (0)
Rash	8 (30.7)	3 (11.5)	5 (19.2)	0 (0)
Paronychia	1 (3.8)	0 (0)	1 (3.8)	0 (0)
Hand-foot syndrome	5 (19.2)	1 (3.8)	4 (15.4)	0 (0)
Hematologic abnormality	3 (11.5)	0 (0)	1 (3.8)	2 (7.7)
GI bleeding	2 (7.7)	0 (0)	2 (7.7)	0 (0)
Hypothyroidism	3 (11.5)	3 (11.5)	0 (0)	0 (0)
Mucosal inflammation	5 (19.2)	3 (11.5)	2 (7.7)	0 (0)
Hemorrhagic cystitis	1 (3.8)	1 (3.8)	0 (0)	0 (0)
Dyspnea	2 (7.7)	2 (7.7)	0 (0)	0 (0)
Sonar	2 (7.7)	2 (7.7)	0 (0)	0 (0)
Tinnitus	1 (3.8)	1 (3.8)	0 (0)	0 (0)
